# Early processes in heme-based CO-sensing proteins

**DOI:** 10.3389/fmolb.2022.1046412

**Published:** 2022-11-03

**Authors:** Marten H. Vos, Mayla Salman, Ursula Liebl

**Affiliations:** Laboratoire d'Optique et Biosciences, Centre National de la Recherche Scientifique, Institut National de la Santé et de la Recherche Médicale, École Polytechnique, Institut Polytechnique de Paris, Paris, France

**Keywords:** carbon monoxide, heme, sensor protein, ultrafast spectroscopy, ligand dynamics, CooA, RcoM

## Abstract

Carbon monoxide has been recognized relatively recently as signaling molecule, and only very few dedicated natural CO sensor proteins have been identified so far. These include in particular heme-based transcription factors: the bacterial sensor proteins CooA and RcoM. In these 6-coordinated systems, exchange between an internal protein residue and CO as a heme ligand in the sensor domain affects the properties of the DNA-binding domain. Using light to dissociate heme-ligand bonds can in principle initiate this switching process. We review the efforts to use this method to investigate early processes in ligand switching and signaling, with an emphasis on the CO-“trappingˮ properties of the heme cavity. These features are unusual for most heme proteins, but common for heme-based CO sensors.

## 1 Introduction

Carbon monoxide is an inert diatomic gas molecule. Atmospheric CO results from the incomplete combustion of carbon sources in natural and industrial processes (Raub et al., 2000), whereas in mammalian cells CO is generated predominantly during the degradation of heme by heme oxygenases (Soares and Bach, 2009). CO is toxic at high concentrations due to its very efficient binding to oxygen binding sites in heme proteins, including those responsible for oxygen transport and storage, such as hemoglobin and myoglobin, respectively, and its inhibition of respiratory oxidases. On the other hand, at low concentrations, CO is now also recognized as an important signaling molecule (Boczkowski et al., 2006; Gullotta et al., 2012; Heinemann et al., 2014; Hopper et al., 2020; Siracusa et al., 2021). In mammalian systems, at physiological levels, CO can mediate various signaling processes, including the production of inflammatory mediators (Otterbein, 2002), apoptosis (Almeida et al., 2012) or signaling in the central nervous system (Queiroga et al., 2015), and can have an antimicrobial function (Chin and Otterbein, 2009). Several bacterial species can utilize CO as energy source, and CO oxidation systems are present in a number of microbes (Diender et al., 2015; Robb and Techtmann, 2018). Studies on the bactericidal mode of CO action showed that CO decreases the respiratory rates due to its direct binding to terminal oxidases (Davidge et al., 2009; Smith et al., 2011) and maintains its bactericidal properties under anaerobic conditions (Nobre et al., 2007). In order to keep the cellular CO concentration below toxic levels, several bacterial species rely on detection and binding of CO by specific sensor proteins. To date, only a very limited number of proteins have been identified that exclusively serve as direct CO sensors. All of them are heme proteins and part of a group of allosteric proteins that undergo switching heme configurations and CO-induced ligand displacement, which induce modifications in a distant protein domain and its associated function (Shimizu et al., 2015; Négrerie, 2019). These CO sensors include the bacterial single component transcription factors CooA and RcoM that contain an N-terminal heme-binding and a C-terminal DNA-binding domain. CO binding to the heme sensor domain triggers conformational changes that allow protein binding to their target DNA sequence and eventually upregulation of the transcription of the *coo* and *cox* gene products, respectively, which catalyze conversion of CO to CO_2_ (Roberts et al., 2004; Mendes et al., 2021). Mammalian proteins containing heme domains have also been shown to be CO-responsive, including the NPAS-2 transcriptional regulator associated with circadian rhythm control (Dioum et al., 2002), cystathionine β−synthase (Banerjee and Zou, 2005) and several ion channels (Wilkinson and Kemp, 2011; Peers et al., 2015), but the effective functioning of these systems as CO sensors is still not fully established.

**FIGURE 1:**
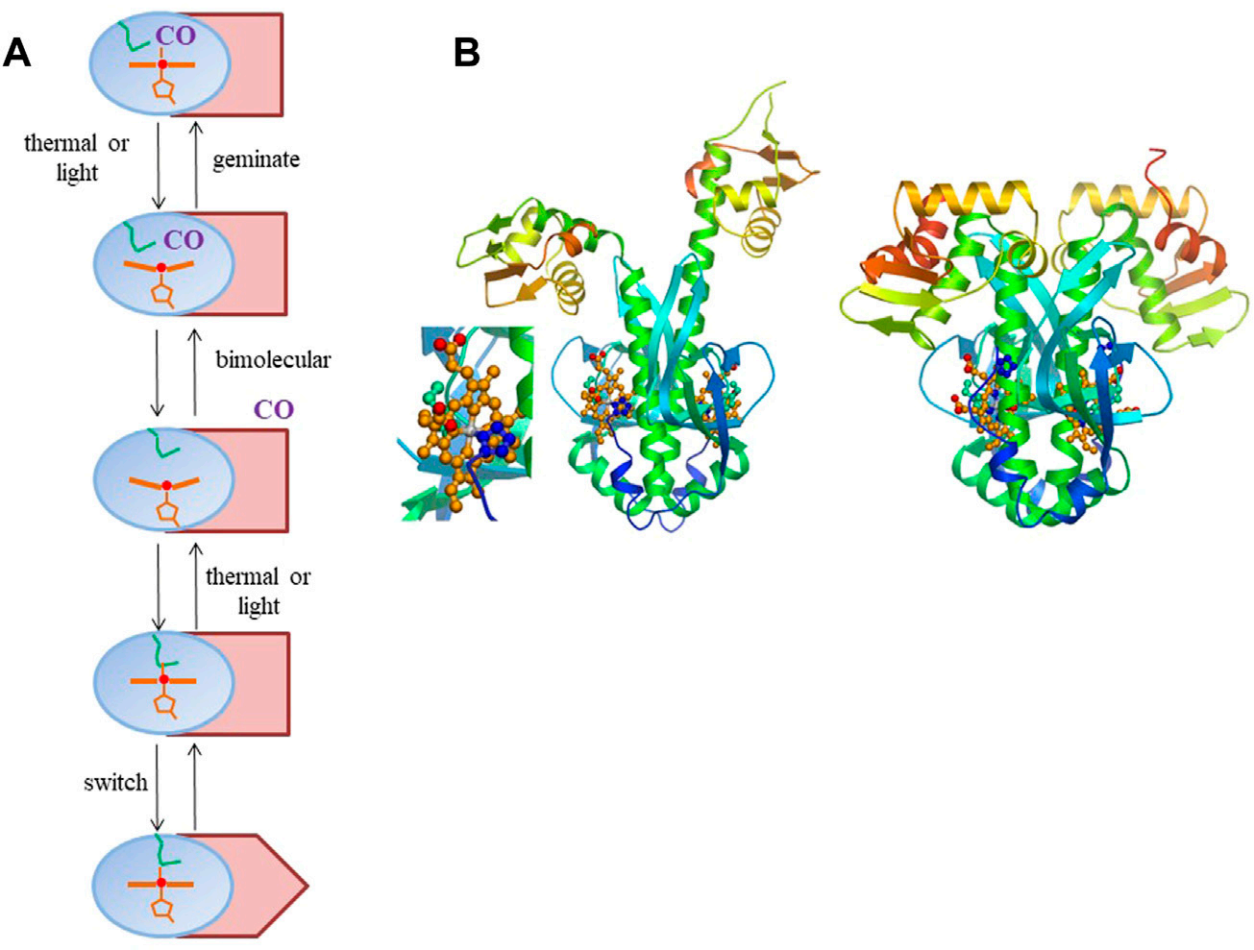
**(A)** General scheme of intra-protein signal transmission in heme-based CO sensor proteins where CO and an internal residue (here depicted by a methionine, as in RcoM sensor proteins, green). The heme domain is in blue; the DNA-binding domain in Indian red, with only the rectangular configuration allowing DNA binding. The scheme is a minimal scheme, for instance not including potential hysteresis effects in back switching and cooperative effects within homodimeric proteins. **(B)** Model for *R. rubrum* CooA structural changes upon CO binding based on the crystal structure of the of the inactive form (Borjigin et al., 2007) (right, with close-up of the heme-proline bond) and the crystal structure of the *C. hydrogenoformans* imidazole-bound form (Komori et al., 2007) For details see (Liebl et al., 2013). Panel b reprinted from Biochimica Biophysica Acta—Proteins and Proteomics, 1834, U. Liebl, J.-C. Lambry, M. H. Vos, Primary processes in heme-based sensor proteins, 1,684–1,692, Copyright (2013), with permission from Elsevier.

In heme-based CO-sensor proteins, the pathway of allosteric change involves exchange of an internal (amino acid) heme ligand and CO, both stable switching configurations being six-coordinate (Figure 1A). This contrasts with the mammalian NO receptor guanylate cyclase and its bacterial analogues, where the stable switching configurations are both 5-coordinate, with either NO or a histidine residue as ligand, and where the moderately responsive CO-bound form is also 6-coordinate (Derbyshire and Marletta, 2012).

In the present mini-review we will mainly focus on the two unambiguously identified bacterial systems. As mentioned above, in these heme-based sensor proteins, CO interacts with the heme iron and its binding to the heme Fe(II) complex changes the heme coordination state. Establishing the intra-protein pathway in which heme coordination changes lead to functional changes in the remote DNA-binding effector domain is of high interest. For the structurally best characterized sensor CooA, this has been studied for instance using structural modeling (Borjigin et al., 2007), structural and spectroscopic (Resonance Raman) approaches on CooA constructs that lack the DNA binding domain (Ibrahim et al., 2007) or are locked in one configuration (Tripathi and Poulos, 2018), or in which the conformational flexibility of the (non-structurally characterized) oxidized protein is evaluated by steady-state site-directed spin-labeled EPR spectroscopy (Hines et al., 2018).

In principle, time-resolved spectroscopic or structural methods may allow characterizing functional switching intermediates and pathways following dissociation of the heme-ligand axial bond upon light absorption by the heme. Although this photochemical phenomenon does not correspond to any solidly documented functional property, it is widely used to provoke heme-ligand dissociation using short light pulses, down to the femtosecond timescale, during time-resolved optical spectroscopy experiments (Vos, 2008). In this way, photodissociation mimics the natural stochastic thermal bond-breaking events in a synchronized way (Figure 1A). Furthermore, making use of the fact that heme ligation and redox states can be easily monitored in their visible absorption spectrum, the dynamics of heme proteins, and in particular that of the prototypical heme protein myoglobin, a small oxygen storage protein, have been extensively studied. Specifically, after photodissociation, the migration and rebinding dynamics of external diatomic gas ligands (CO, NO, and O_2_) or internal amino acid residues can be followed.

In non-sensor heme-proteins like myoglobin and hemoglobin, photo-dissociated CO [photochemical quantum yield close to unity (Ye et al., 2002)] initially remains in the heme pocket, but does not rebind to the heme in a geminate process^1^ to a significant extent, and ultimately escapes from the protein with high yield (Table 1). If the same would hold true for heme-based CO sensor proteins, this would provide a relatively straightforward method to study intermediates in signal transduction from the heme sensor domain to the DNA binding effector domain in these proteins. However, as discussed below, this is generally not the case, as high-yield heme-CO geminate rebinding occurs. This is in general agreement with observations of strong and presumably functionally relevant geminate rebinding of the physiologically sensed ligand in other heme-based ligand sensor proteins (Négrerie et al., 2001; Liebl et al., 2002; Liebl et al., 2013). This mini-review focusses on spectroscopic studies of the early processes occurring on the timescale of the rebinding processes, illustrating their possibilities and limitations, and discussing their relevance.

**TABLE 1:** Time constants τ and corresponding relative amplitudes *A* of CO recombination kinetics in various CO sensor protein constructs and CO-responsive proteins, as well as myoglobin; the description in terms of multi-exponential decay functions reflects underlying heterogeneity in the kinetics, but not necessarily distinct decay phases.

Protein	Species	τ_*1*_ (ps)	*A*_*1*_ (%)	τ_*2*_ (ps)	*A*_*2*_ (%)	*τ*_*3*_ (ps)	*A*_*3*_ (%)	*A*_*∞*_ (%)	Reference
CooA	*R. rubrum*	78	60	368	30			10	Kumazaki et al. (2000)
RcoM-2 heme domain	*B. xenovorans*	170	65	500	35			0	Bouzhir-Sima et al. (2016)
RcoM-2 full length	*B. xenovorans*	180	45.3	660	54.1			0.6	Salman et al. (2019)
Cor	*M. tuberculosis*	60	45	440	34	>7,000	20		Salman. (2019)
SUR2A	rat	22	14	150	26	2,500	50	10	Kapetanaki et al. (2018)
myoglobin	horse	160.000	5.5					94.5	Cao et al. (2004)

## 2 CooA

The best characterized heme-based CO sensor protein is the anaerobic CO-activated transcriptional activator CooA. It is found for instance in the photosynthetic purple bacterium *Rhodospirillum rubrum*, where it activates the transcription of genes required for the anaerobic oxidation of CO to CO_2_ (Roberts et al., 2005). CooA has a micromolar affinity for CO and binds CO only when its heme is in the Fe^2+^-state, whereas oxidized CooA is not CO-responsive. CooA is a homodimer, with each subunit comprising a heme-binding domain and a DNA-binding domain. CO-binding induces conformational changes that allow CooA to bind to target DNA and activate transcription of *coo* genes. This is not the case for O_2_, which leads to autoxidation, or NO, which leads to formation of a pentacoordinate nitrosyl complex that is not directly ligated to the protein any more (Ascenzi et al., 2004). The CO-induced switching of CooA from the inactive to the active form presumably encompasses substantial conformational changes, as indicated by the crystal structure of the inactive form and structural modeling of the active CO-bound form (Borjigin et al., 2007; Liebl et al., 2013) (Figure 1B). In either active or inactive steady-state configuration, the heme is always 6-coordinate, with the switching being triggered by exchange of an internal protein ligand and the external CO molecule. Remarkably, in the inactive ferrous form, the heme is coordinated by a histidine from one subunit and an N-terminal proline residue (Pro-2) from the other subunit. In virtually all other 6-coordinate ferrous proteins axial heme coordination occurs by histidine or methionine.

As indicated above, switching between the active and inactive forms may be initiated by photo-dissociation, in principle either of the internal amino acid ligand or the CO-ligand. Excitation of the fully-reduced enzyme in the absence of CO indeed leads to dissociation of the proline-2 ligand. Despite its position at the N-terminus, this dissociation nevertheless leads to virtually complete rebinding in ∼6.5 ps (Kumazaki et al., 2000) and not to a long-lived dissociated state. Such rebinding of internal ligands in a few picoseconds is a general feature for reduced 6-coordinate heme proteins liganded by two amino acids, including for other heme-based sensor proteins (Vos et al., 2008; Liebl et al., 2013).

The (cooperative) CO binding to the CooA dimer occurs with modest affinity, with Km values in the order of 1 μM (Puranik et al., 2004). Excitation of the CO-bound form dissociates the CO from the heme. Subsequently, the vast majority (>90% at physiological temperatures) of dissociated CO rebinds on the tens and hundreds of picoseconds timescale in a multiphasic way (Table 1), as assessed by time-resolved visible (Kumazaki et al., 2000; Lobato et al., 2014; Benabbas et al., 2017) and Raman (Uchida et al., 2000) (heme coordination) spectroscopy, as well as by time resolved infrared spectroscopy (Rubtsov et al., 2001) (CO coordination). At the time, this was by far the most extensive CO geminate recombination observed in any heme protein. This CooA property is most likely due to a constrained heme pocket, in which the dissociated CO cannot easily rotate and remains close to perpendicular to the heme plane; an orientation allowing (re-) binding to the heme. This situation contrasts markedly with the case in myoglobin, where dissociated CO rotates, on the femtosecond timescale, to a position more parallel to the heme plane, which effectively hinders rebinding, although the dissociated CO remains very close to the heme iron (Lim et al., 1997). Interestingly, in general agreement with this reasoning, in a CooA mutant protein, where the proximal histidine is replaced by glycine, a much larger fraction of CO remains dissociated. This fraction corresponds to a conformation in which CO is initially bound under an angle of > 20°C with the heme normal (Zhang et al., 2006), which may hinder rebinding. Polarized infrared transient absorption experiments, including on the non-bound CO (present experiments on CooA are limited to the heme-liganded CO, which has higher extinction) may shine further light on the mechanism underlying the fast recombination of the heme-CO bond.

The very high CO geminate recombination yield complicates the use of flash photolysis to study the switching mechanism directly (see below). Notwithstanding this limitation, the kinetics can give more indirect information on allosteric interactions allowing switching. The heterogeneity in the rebinding kinetics has been ascribed to conformational heterogeneity of the heme pocket (Benabbas et al., 2012; Benabbas et al., 2017). Binding of the CooA DNA-binding domain to its target DNA renders the CO rebinding kinetics less heterogeneous, suggesting rigidification of the heme-binding domain (Benabbas et al., 2012). Thus, allosteric interactions between heme-binding and DNA-binding domains are bidirectional. The fraction of CO that can escape from the heme pocket also further decreases in the presence of target DNA (Benabbas et al., 2012), suggesting an influence of the DNA itself on the switching frequency.

Whereas time-resolved experiments on ultrafast timescales necessarily are performed under low excitation conditions (<< 1 excitation/heme), on longer time scales longer and intense excitation pulses can be used that allow multiple excitations [i.e., re-dissociations after geminate recombination of dissociated heme-CO pairs, cf. (Liebl et al., 2003)]. Under the latter conditions the net dissociation per pulse is increased, yielding higher transient long-lived signals. This approach has been employed on (DNA-devoid) CooA by Spiro and coworkers performing microsecond time-resolved transient absorption and transient Raman spectroscopic studies (Puranik et al., 2004). They assessed that proline-binding after CO dissociation from the protein occurs on the millisecond time scale and that further conformational changes occur on the seconds and even minutes time scale.

## 3 RcoM

In 2008, a second class of bacterial heme-based CO-responsive transcriptional regulators, RcoM, was discovered in the heterotrophic aerobic bacterium *Burkholderia xenovorans* that is capable of oxidizing carbon monoxide (Weber and King, 2012). It consists of two highly homologous heme proteins, RcoM-1 and RcoM-2 (Kerby et al., 2008) that were recently shown to be homodimeric (Dent et al., 2022), with each monomer harboring a heme-binding PAS domain and a DNA binding LytTR domain. Purified RcoM-1 binds CO with very high affinity and exhibits low-affinity DNA-binding, and acts as a transcriptional switch that senses low but persistent CO levels (Kerby and Roberts, 2012). The sensor-heme of the fully reduced forms of RcoM proteins is ligated by histidine and methionine (Marvin et al., 2008; Bowman et al., 2016), with the latter being exchanged by CO upon CO exposure. Although structural information on these proteins is still lacking, they have some very remarkable properties that are clearly distinct from CooA. First, the CO affinity for RcoM proteins was found to be extremely high, to a point that constructs of the protein can be, under aerobic conditions, purified in the CO-bound form (Kerby et al., 2008; Bouzhir-Sima et al., 2016). For full length RcoM-1 (Kerby and Roberts, 2012) and RcoM-2 (Salman et al., 2019) it is a few nM; in the isolated RcoM-2 heme domain (RcoMH-2) even subnanomolar (Bouzhir-Sima et al., 2016). Furthermore, and probably related, upon CO photodissociation, geminate heme-CO recombination occurs to an even higher extent than in CooA, virtually complete (>99.5%) for the isolated RcoM-2 heme domain and at 99.5% for the full-length protein (Bouzhir-Sima et al., 2016; Salman et al., 2019) (Table 1). These are unprecedented properties for any heme protein that likely determine the low (thermal) CO dissociation rate, as they imply that any CO that is thermally dissociated from the heme can hardly escape from the protein. The RcoMH-2 construct (CO dissociation time constant ∼80 h) may therefore be used as an efficient quasi-irreversible CO scavenger.

In RcoM-2 constructs, the CO rebinding kinetics are multiphasic, as in CooA, but somewhat slower, with time constants only in the hundreds of picoseconds timescale. The presence of the DNA-binding domain substantially slows down the kinetics (and allows a small but measurable asymptotic value corresponding to CO escape; see above), pointing at bidirectional allosteric interactions between the two domains (Salman et al., 2019).

Furthermore, upon addition of DNA to full-length RcoM-2, a very small but sizeable acceleration of the rebinding occurred, a qualitatively similar effect as observed in CooA (see above), and also ascribed to a rigidification of the heme domain (Salman et al., 2019). Yet, very recently, an in-depth investigation into the binding of the DNA sequence to RcoM-2 used in this study concluded against specific binding (Dent et al., 2022), suggesting this effect might have been due to non-specific binding.

To date, no attempts to study processes occurring on longer time scales after CO dissociation from RcoM proteins have been reported, presumably related to the prohibitively low escape rate of CO. Efforts to understand the molecular switching mechanism, which might be studied in real time on the timescale >∼100 ms by mixing techniques starting from the fully reduced system (Bouzhir-Sima et al., 2016), must further await structural characterization.

## 4 Other systems

For completeness we briefly mention work on other (potential) CO sensors. First, the product of the *cor* (*CO resistance*) gene is strongly implicated in the sensitivity of mycobacteria for CO (Zacharia et al., 2013) and therefore of potential therapeutic interest. Expressed *Cor* protein was found to be able of heme binding, and of binding CO *via* this heme (Salman, 2019). Strongly multiphasic heme-CO geminate recombination on the tens of picoseconds to nanoseconds timescale occurs in this system (Table 1), but with a substantially higher CO escape yield (∼20%) than the systems discussed above. This property may allow more extensive mechanistic studies using spectroscopic techniques once the protein has been characterized in more detail. Similarly highly multiphasic rebinding kinetics were observed in CO recombination to heme in the CO-responsive mammalian K_ATP_ channel SUR2A (Kapetanaki et al., 2018) (Table 1). Altogether, the multiphasic CO rebinding kinetics found in the ensemble of CO-responsive proteins point at a common high flexibility of the CO-bound form, which may have functional relevance as alluded to in Section 3 for CooA.

The response of CO dissociation in other interesting mammalian CO-responsive proteins remains to be studied. For instance, human cystathionine β-synthase catalyzes the formation of cystathionine from serine and homocysteine. The enzyme contains an N-terminal heme-binding domain, a catalytic core and a C-terminal regulatory domain, and catalysis is regulated by heme redox state and CO binding (Banerjee and Zou, 2005; Singh et al., 2007). The enzyme containing a very stable heme Fe(III) complex is active, whereas CO (or NO) binding to the heme Fe(II) complex inhibits catalysis. Although it has been postulated that NO and CO might act together as physiological gas signaling molecules in enzyme function (Vicente et al., 2014; Shimizu et al., 2015), as they bind only to the Fe(II) complex, this raises questions about the feasibility of forming a heme Fe(II)-CO/NO complex under physiological conditions. In this context it is interesting to mention Rev-Erbβ, a nuclear receptor that couples circadian rhythm, metabolism and inflammation. Heme-binding modulates its repressor function and its activity in gas sensing. Rev-Erbβ binds Fe(III)-heme (which is crucial for its repressor activity) more tightly than Fe(II)-heme. This might appear difficult to reconcile with Rev-Erbβ′s role as a CO/gas sensor, which can only bind to Fe(II). Very recently it has been shown that Fe(III)-Rev-Erbβ remains heme-bound and undergoes reduction in the presence of diatomic gases, in line with a CO/gas sensing function (Sarkar et al., 2021).

## 5 Concluding remarks

A common feature emerging for all CO sensor and CO-responsive protein systems studied is the very high yield of geminate rebinding, so that they effectively function as a “CO trapˮ, in which CO dissociated from the heme (whether thermally, as occurs physiologically, or by light) can hardly escape the heme pocket. On the molecular level, this property is unusual for CO, as its binding to heme requires near-perpendicular orientation of the heme-CO system. Thus dissociated CO is expected to have very limited conformational freedom in the heme pockets of these sensors. Further transient infrared and ultimately transient crystallographic studies may clarify the molecular mechanisms underlying this property. On the functional level, these properties help to reduce effective CO dissociation rates and therewith adapt the response time of the sensor to physiologically relevant times, smoothening out the effect of fast environmental fluctuations (Liebl et al., 2013). In this context, it is interesting to note that low escape yields of the sensed ligand are more general properties for heme-based gaseous ligand sensors, including the O_2_ sensor FixL (Liebl et al., 2002) and the NO receptor guanylyl cyclase (Négrerie et al., 2001).

## Footnotes

1 In *geminate* rebinding the same pair of molecules that were dissociated reform a bond; this process occurs with rates that are independent of the ligand concentration in solution. In contrast in *bimolecular* (re)binding, a molecule (here CO) from the solvent binds with a dissociated counterpart (here heme) with a rate that increases with the ligand concentration in solution.
